# A protocol for retrospective translational science case studies of health interventions

**DOI:** 10.1017/cts.2020.514

**Published:** 2020-07-22

**Authors:** Sara E. Dodson, Ira Kukic, Linda Scholl, Clara M. Pelfrey, William M. Trochim

**Affiliations:** 1 Office of Science Policy and Planning, National Institute of Neurological Diseases and Stroke, National Institutes of Health, Department of Health and Human Services, Bethesda, MD, USA; 2 Office of Evaluation, Performance and Reporting, Division of Program Coordination, Planning, and Strategic Initiatives, National Institutes of Health, Department of Health and Human Services, Bethesda, MD, USA; 3 Office of Applied Scholarship and Education Science, Mayo Clinic College of Medicine and Science, Rochester, MN, USA; 4 Clinical and Translational Science Collaborative, Case Western Reserve University, Cleveland, OH, USA; 5 Clinical and Translational Science Center, Weill Cornell Medicine, Cornell University, New York, NY, USA

**Keywords:** Mixed-method case study, impact, translational research evaluation, translational science, health intervention

## Abstract

The critical processes driving successful research translation remain understudied. We describe a mixed-method case study protocol for analyzing translational research that has led to the successful development and implementation of innovative health interventions. An overarching goal of these case studies is to describe systematically the chain of events between basic, fundamental scientific discoveries and the adoption of evidence-based health applications, including description of varied, long-term impacts. The case study approach isolates many of the key factors that enable the successful translation of research into practice and provides compelling evidence connecting the intervention to measurable changes in health and medical practice, public health outcomes, and other broader societal impacts. The goal of disseminating this protocol is to systematize a rigorous approach, which can enhance reproducibility, promote the development of a large collection of comparable studies, and enable cross-case analyses. This approach, an application of the “science of translational science,” will lead to a better understanding of key research process markers, timelines, and potential points of leverage for intervention that may help facilitate decisions, processes, and policies to speed the sustainable translational process. Case studies are effective communication vehicles to demonstrate both accountability and the impacts of the public’s investment in research.

## Introduction

The science of translational science seeks to understand the scientific and operational principles underlying each step of the translational research process [1]. While the translational process is not linear, several distinct phases of research are typically operationalized, for example, basic, preclinical, clinical, clinical implementation, and public health research, with critical translation efforts required to move knowledge between each phase. To systematically assess the complex translational process, several promising formative and summative research evaluation approaches, including quantitative, qualitative, and mixed methodologies, have been developed in recent years [2,3]. Case studies frequently are used as tools for research evaluation because they provide a rigorous way to explain understudied practices, and they are an effective mechanism for identifying long-term outcomes of scientific research [4–7]. In addition, researchers who study the key processes and outcomes of scientific endeavors are continually refining frameworks for assessing scientific research impact [6,8–14].

A systematic translational science case study approach is currently lacking. This article fills this gap by providing a specific protocol for conducting case studies to evaluate the translational research processes underlying the development of successful health interventions. This protocol allows researchers to apply a common approach and generate comparable insights. The authors recommend Robert Yin’s textbook “Case Study Research and Applications: Design and Methods” for a full exploration of the theoretical foundations of the case study methodology [15]. Yin describes how case studies are an excellent evaluation tool as they allow for the combined use of qualitative and quantitative data, providing an in-depth examination of the factors that contributed to the success of specific research activities, as well as factors that may hinder success [15–18]. The process of conducting case studies requires an open and flexible approach that is driven by the unique case being studied. Case studies can capture a wide variety of impacts, including the unexpected, and can provide context about the evolution of research that may not be apparent in a review of outcomes. Case studies are particularly valuable in describing whether and how certain activities and contributors were pivotal in advancing science and improving public health outcomes [6,19]. Translational case study researchers often face the challenge of keeping the case focused on a specific and time-bound translational intervention and its evolution. Case study researchers must continually make decisions about which elements are and are not fundamental to the story.

Case studies, like all methodologies, have limitations. Results from a single case may not be generalizable [18]. Our case study protocol is not meant to cover the full scope of a research program, but rather to capture the central elements in the discovery and development of a specific health intervention. As such, it may potentially filter out aspects of a larger field of research in which the case is situated. In addition, case studies are data-intensive, time-consuming, require expert input to highlight the most important factors, and are susceptible to subjective interpretation [20]. Despite such limitations, case studies are arguably the most comprehensive way to study complex systems.

The sections below provide guidance on selecting cases for study; the key elements, themes, and analyses that are needed to develop the cases; as well as methods and data sources useful in conducting case studies. We encourage adoption of this protocol by diverse researchers working in any number of fields who focus on understanding the scientific process and research outcomes. The case study approach is most effective when findings are made accessible to broad communities, in particular because successful translation is often impeded by a lack of common vernacular between research disciplines, practice communities, and policymakers. Ultimately, the goal is to develop a collection of comparably-conducted case studies, enabling cross-case analyses that could inform the “science of translational science” to address questions such as: What processes tend to drive translation forward and in which contexts? What challenges can be anticipated with particular types of research studies? How can such challenges be addressed early to avoid delays in successful implementation? How can resources from research institutions and funders be directed to maximally support translational research efforts?

## Translational Case Study Protocol

### Case Selection

Appropriate case types include evidence-based interventions which have generated discernible health impacts, such as a specific technology, diagnostic, preventive, drug, device, biologic, behavioral intervention, or other treatment strategy. Cases should be examples of successful translation across the full continuum of research to practice, where the generation of knowledge falls within a definable range between *inception of the intervention* and *its impacts*. The selected intervention should be currently in use in medical or public health settings and there should be evidence that the intervention improves health outcomes, increases life expectancy, and/or improves individuals’ quality of life.

Case studies of research translation that have not progressed yet to impact, but which show strong potential for future impact, are also valuable, as are studies that examine “unsuccessful” aspects of research translation. However, unsuccessful research is much harder to study because there are very few negative studies published, and researchers are reluctant to highlight their failures. While this protocol may be adapted to the study of partial and not-yet-successful research translation efforts, the focus here is on the assessment of interventions that have been successfully implemented into practice.

### Case Study Elements

The case study consists of two central elements: (1) a detailed timeline of the major events and milestones that marked the translational progress, and (2) a broader narrative that describes how and why such progress happened. The timeline and narrative should complement each other and contain overlapping content. Both should include detailed documentation of sources that support the central elements. A more detailed description of the translational case study elements is presented in Appendix A (in Supplementary Material).

#### The case study timeline

Case studies should include a timeline, or multiple interconnected timelines where warranted, which serves as a key graphic for organizing and communicating the case’s central information. The timeline is a universally understood device for visualizing temporal translational progression, for example, “distance” between milestones and complex cause and effect relationships. The timeline progresses in phases along one or more pathways and is punctuated by multiple milestones that help to anchor the case study’s chronological story. While timelines are typically linear, we recognize that the translational process often moves backward and forward through different phases and may have parallel storylines. A timeline is particularly effective for describing such parallel storylines and illustrating critical points of convergence and divergence. Organizing elements of the timeline include the following:

1. *Start and end points of case study*: Translational case studies link the chain of evidence from scientific observations to verifiable impacts of the intervention on health. The discreet start and end points of the case should be identified and a rationale for why those points were chosen should be presented. See Appendix A for a detailed discussion and guiding questions on how to select appropriate start and end points. Briefly, selecting the most relevant and appropriate start and end points for any translational case will be subjective and may be challenging. The start point is typically defined as the inception of the particular innovation being described in the case study and its association with a “target” such as a disease or diagnosis. Discussion of the start point chosen for the case study may point to foundational research knowledge that was essential for conceptualizing an effective intervention, perhaps going back decades or more in the research literature. The case study should address how far into practice the intervention has gone and the end point should represent a concrete outcome that has taken place in medical or public health practice. Outcomes may include how the intervention was implemented or otherwise “packaged” for scaling up. If the intervention is a drug, device, or biologic, evidence of adoption into practice should be included, if available. The development and adoption of an intervention in clinical and community settings may continue to evolve far beyond the chosen end point of the case study. When relevant, the start and end points need to be described in the larger context of scientific progress and may require additional relevant historical, social, and political context.
2. *Progress markers/milestones*: Markers or milestones are integrally related to key events that occur during the translational process, including the start and end points of study [21]. Markers are anchored on specific dates and can be represented as points or intervals on a timeline. Markers should be chosen for their ability to help tell the story of the development and translation of the intervention. Different types of markers include the following: (1) major inputs – resources, human and intellectual capital, and so on; (2) key activities and events – major meetings, formation of a collaboration or partnership, serendipitous events, interim research milestones, and so on; and (3) major products or outputs – presentations, publications, clinical trials results, drug approvals, markers of commercialization, changes in practice, changes in public health measures, and any other evidence of adoption of the intervention into practice.
3. *Translational phases*: It is useful to group a complex translational timeline visually into general research phases. Numerous similar multiphase schemes of translation have been proposed, but there is currently no universally accepted typology [21]. One model for these case studies is the National Institutes of Health (NIH) National Center for Advancing Translational Sciences’ translational phase model [22], which includes the following research phases: basic, preclinical, clinical, clinical implementation, and public health. Another useful translational research framework comes from the NIH’s National Institute of Environmental Health Sciences (NIEHS) [23]. The NIEHS framework was developed specifically to aid researchers in describing the evolution of their translational research in the area of environmental health. See Appendix B (in Supplementary Material) for suggested definitions and parameters for delineating research phases. Depending on the case, it may be necessary to apply a different translational model; however, whatever schema is used, it should clearly identify the markers and milestones that distinguish each phase. Well-written case studies should help reveal where there are intersecting points and gray areas between the discrete phases.

#### The case study narrative

The second central element of the case study is the narrative, which provides a coherent summary that moves the reader through the translational science process, describing the major actors, themes, forces, pivotal events, and advances that influenced the translational process. The narrative should focus on describing *how and why* the intervention developed as it did, *how and why* the markers/milestones were achieved, as well as what challenges were encountered and how those challenges were addressed. These drivers of translation may arise directly from key documents and/or interviews with the central researchers and stakeholders (e.g., funders, community advocates, or practitioners). They may also arise indirectly through an analysis of the information gathered throughout the course of researching the case study. Given the interdisciplinary nature of translational research, the narrative should avoid discipline-specific jargon and instead should use easily understood language. In addition, case study authors may want to consider writing the narrative for different target audiences, including a lay audience (see section “Formats of Finalized Case Study Materials” for a broader discussion of case study audiences and formats).

#### The case study narrative key elements

1. *Health problem and relevance of the intervention*: The case study narrative should begin with: (a) background on the relevant disease(s), disorder(s), or public health challenge(s), including some measure(s) of burden to help communicate the scope of the problem; (b) a description of the intervention; (c) relevant historical, social, and political context; and (d) a brief summary of the impact.
2. *Key events:* The key events are the scaffolding of the case study narrative and often correspond with the timeline progress markers/milestones, including the start and end points. They constitute the heart of the chronological story, describing the sequence of integral events.
3. *Key people and partnerships*: Over the course of translating an intervention from inception to impact, there are many individuals and groups who play important roles in the research progress. In a case study, determining which key actors are discussed, and why, requires careful judgment and should be backed by objective evidence. The case study should highlight individuals and sectors across the health research and practice ecosystem. This should include the central researchers and teams as well as those who were integral in disseminating and implementing the intervention, in the commercial or nonprofit development of the intervention, and in enabling broad uptake and adoption of the intervention. In addition, there should be a description of how and why different individuals collaborated with each other and what role those collaborations played in the development and implementation of the intervention. Collaborative relationships are often influenced by surrounding organizational culture(s) in ways that may be conducive or disruptive to the success of collaborative research endeavors. Where relevant, consider examining the characteristics of the organizational climate that helped create and support key collaborations.
4. *Other influencing factors:* There are many other factors that can influence a translational research process. Case studies should include descriptions of major facilitators and barriers, both expected and unexpected. Facilitators may include critical support and infrastructure; influential policies; transformative technologies, tools, and techniques; and knowledge or strategies borrowed from tangential lines of research. Major barriers or challenges should also be described, including failed or abandoned research directions, and how those difficulties were overcome. In addition, there may be critical contextual factors that influenced translational progress, such as historical, political, and other social events or changes. Well-designed interview questions are particularly useful to draw out influential factors that may not be obvious to those centrally involved in the research nor readily apparent from records and other archival materials.
5. *Impacts*: The case study should provide evidence, and whenever possible, a graphical display that conveys a current “snap-shot” of realized impacts. As noted in the introduction, there are several frameworks and metrics for assessing scientific research impact [6,8–14]. One extensive and useful community resource is the Becker Medical Library Model for Assessment of Research Impact [10,24]. Drawing from these frameworks, we advise identification of three distinct categories of impacts: impacts on health – for example, changes in health outcomes at the individual and population level; scientific knowledge impacts – for example, emergence and growth of new fields, improved methodological and technological tools/applications; and other societal impacts – for example, cost savings, economic activity/growth, human and intellectual capital, improvements in science and health literacy. Minimally, clear evidence of impacts on health is expected for any complete translational cases. See Appendices A and B for a more detailed descriptions of diverse impacts that case study researchers could examine.
  Impacts will rarely be fully attributable to the case study; in most cases, they will be influenced by many additional moderating factors not covered in the study. Therefore, case study researchers should avoid “over-crediting” their findings, and should provide compelling evidence that the central factors identified have played a critical role.
6. *Further developments*: Case study narratives should conclude with a description of how the research, dissemination, and/or implementation is currently progressing (or could progress); analysis of the remaining knowledge gaps and work that still needs to be done; and/or any important postscripts to the case study.

### Translational Case Study Methodology

The methodology for conducting a case study is an iterative process that progressively fills in the case details until no significant additional factors emerge. The methodology described here builds on research approaches used by social scientists, political scientists, historians, and even criminal investigators and investigative journalists. These approaches involve skills that include objectivity, analytical skills, interviewing skills, using mixed-methods, doing literature searches, consulting multiple data sources, and constructing a narrative. The list of methodological steps provided below is not intended to be strictly linear; steps can be revisited as information accumulates. For example, while most case studies will begin with defining the start and end points, these are likely to be revised over time as new information and insights arise. This iterative process allows the timeline to be a key methodological tool to tell the narrative and to identify remaining knowledge gaps.

*Methodological steps may include the following:*

**Fig. 1.**
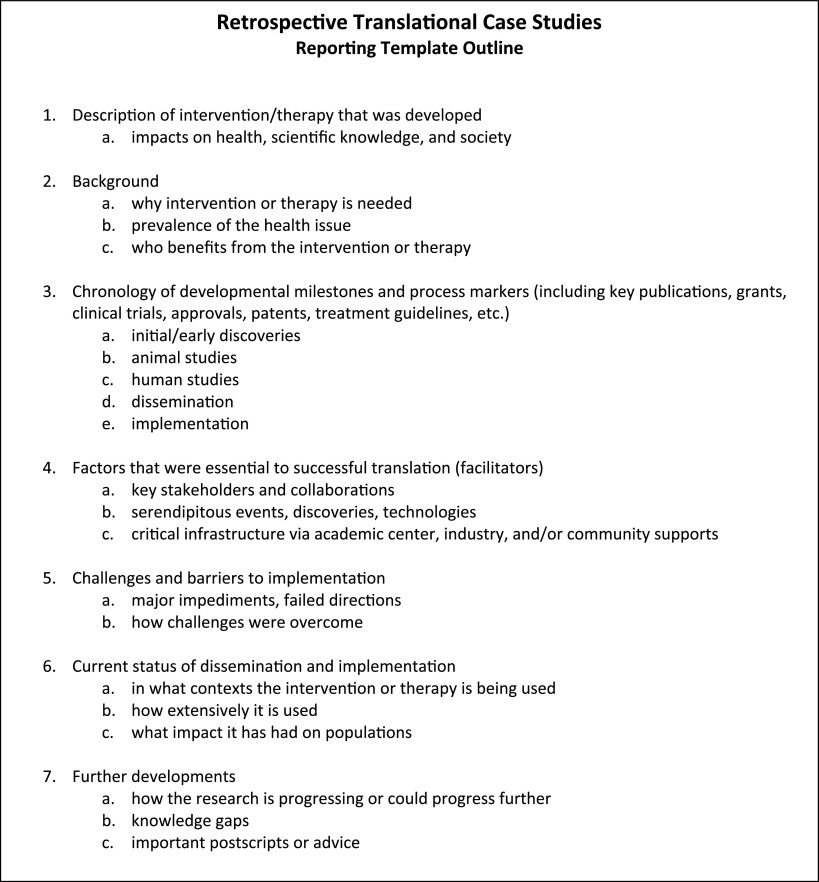
Retrospective translational case study reporting template outline, which can be used as a guide when writing up a case study.

1. Identify and develop:
  a. background on health issue/disease being addressed;
  b. background on key researchers and research team(s);
  c. information about the development, testing, and implementation of the intervention, including key process markers (grants, FDA approvals, clinical trials, patents, publications, research syntheses/meta-analyses, recommendations/guidelines); and
  d. evidence of accrued or potential impacts. Useful information gathering approaches include web searches (including websites maintained by research funders, news media, researchers, industry, health/patient advocacy organizations, professional societies, and so on), literature searches, and other database searches (e.g., for relevant grants, patents, clinical trials, population health data).
2. Create an initial timeline, identifying start and end points and chronologically mapping the progress markers in the translational research process.
3. Identify initial gaps in data/information.
4. Identify an initial list of key stakeholders, including individuals responsible for translational research progress, as well as others who may have strong historical perspective and subject matter knowledge.
5. Conduct semistructured interviews with selected stakeholders.
6. Continue gathering data until no significant additional details or factors emerge.
7. Return case study analyses back to interviewees/key stakeholders to validate and ensure accuracy and completeness.
8. Finalize the case study narrative and timeline (Figure 1).

Data analysis will use a variety of appropriate quantitative and qualitative techniques, which may include:

*Record review*: A variety of records can be used to stitch together the timeline and narrative, including primary and secondary research literature; grant records; press releases and other media items; policy statements; legal and regulatory documents; program and service development announcements; clinical trials; changes in clinical practice guidelines; FDA approvals; patent records; and health service research findings. Often, research and review articles written by the developers of the intervention can be a valuable resource. However, care should be taken to avoid biasing the story toward certain research teams over other potentially pivotal contributors.

*Interviews with key researchers and other stakeholders* are critical for the verification of timeline and story, the identification of central themes and key contextual factors such as facilitators and barriers, and for filling in gaps in the story. Relevant stakeholders include research investigators and trainees, organizational leaders, health and medical practitioners, community health advocates, and patients. The process of conducting semistructured interviews is at the heart of the case study. Wherever possible, it is helpful to develop multiple independent corroborating interviews. Most stakeholders will agree about what the key markers/milestones were; however, there may be competing and irreconcilable stories about how the case evolved. In that situation, alternative stories should be noted rather than forced into a single coherent interpretation. See Appendix C (in Supplementary Material) for key themes to guide the interview and sample interview questions; however, interview guides should be tailored to the case and interviewee. In particular, question probes can be very helpful in encouraging interviewees to place their efforts, understanding, and opinions within the broader context.

*Bibliographic, bibliometric, and grant portfolio analyses* should be used as a rigorous and data-driven approach to identify and validate translational research milestones, central researchers and research funders, levels of research funding, and influential research collaborations. Case study researchers should consider applying these approaches to identify different inputs (such as funding, time, human capital, research infrastructure, equipment/technology, other research resources, and/or partners) as well as different outputs (knowledge generated, patents generated, etc.) that were critical for development of long-term outcomes. A detailed examination of research grant records can provide valuable information on key resources and pivotal research funders. Both the NIH RePORTER webtool [25] and the Federal RePORTER webtool are searchable databases of scientific awards from several federal agencies [25,26].

Relevant to measuring research outputs, bibliometric approaches include several techniques for assessing the quantity, extent of dissemination, and content of publications and patents [2]. For example, bibliographic analysis from an identified clinical trial, grant, or seminal publication can be used to indicate the number of times an article has been cited and in what topic area. The pattern of citations can show the influence of key publications and help provide evidence of knowledge links over time. Network analyses of publication citations can reveal key researchers/research teams, findings, and collaborations. A valuable free bibliometric tool for conducting translational case studies is the “Translational Module” in iCite, a machine learning model that tracks the flow of knowledge into clinical medicine [27,28]. (Details on websites for searching NIH/Federal grants, patents, and bibliometric sources are provided in Appendix B.)

*Review and analysis of health data* should be performed where possible, including review of primary and secondary literature. For example, population health publications and databases can be used to describe changes in disease incidence and severity. In addition, healthcare utilization data may exist (e.g., from enterprise healthcare databases), providing information on health and medical spending. Analyzing federal, state, and health organization policies can help determine how the case may have influenced policy changes.

See Appendix B for a collection of useful data resources organized by research and practice phase as well as type of impact.

### Formats of Finalized Case Study Materials

While the gold-standard for disseminating case study results is publishing in a peer-reviewed research journal, other formats should be considered for dissemination to wider audiences. For example, the NIH has posted several case studies on the Web in an effort to help communicate the value of biomedical research to the general public [29]. These web materials include brief summaries of the broader case study narrative as well as graphics (e.g., stylized timelines, figures, infographics), and detailed documentation and supplemental materials with further information.

There are several potential audiences for these case studies, including various research communities, research funders, health practitioners, domestic and international research policymakers, patient communities, and the general public. While it is encouraged to construct case studies for diverse audiences, findings can also be packaged in a variety of formats aimed at specific audiences. Each of these groups may have different needs, value different aspects of health research, and respond to different types of approaches for disseminating information.

## Future Developments Toward Enhancing the Science of Translational Science

This protocol is intended to be an evolving document, which can be updated and refined as exemplary practices in the science of translational science emerge. This is one of several complementary efforts to enhance the identification of translational science success factors and provide a sustainable and useful framework for both the scholarly and practical study of translational science. Together with this protocol, we encourage the development of (1) appropriate publication outlets, (2) archiving strategies, and (3) coding schemes, to ensure high-quality work that is accessible for further research, including comparative studies.

*Publication outlets and review*: We encourage the development of a special category of publication – the translational research case study – that could be included on an ongoing basis in appropriate journals. We believe a recognized publication mechanism would go a long way toward enhancing the consistency, quality, and accessibility of such reports. Independent peer review of such publications would help assure quality. Conducting case studies is time consuming; therefore, a recognized publication type would help overcome that barrier by providing professional and academic value in the form of creditable publications.
*Coding*: Developing a coding scheme for translational research case studies would enhance their subsequent retrieval and meta-analyses. Each case study could be coded on a number of standard variables, including classification of the type of intervention, disease/disorder/public health research area, populations affected, key markers/milestones, key themes identified, outcomes achieved, and the translational stages covered by the case. No such classification system currently exists, and we should develop one inductively after accumulating a sufficient number of cases. A simple coding/classification scheme would be desirable to include as part of the case study report. Such a scheme would resemble the classifications used to store clinical trials in www.clinicaltrials.gov, such as the Medical Subject Headings (MeSH) coding scheme managed through the National Library of Medicine [30].
*Archiving*: A central archiving repository for translational case studies would provide (1) a certifying mechanism for the quality of cases archived; (2) a motivational mechanism to encourage researchers to contribute to the literature of case studies; and (3) a database to support meta-analyses that could lead to broader generalizations about the factors that influence successful translational research. This repository would include rich meta-data on each study (e.g., coding data described above). Over time, such an archive would help to establish a body of comparable studies supporting research on translation and enable valuable cross-case analyses.

## Conclusions

This protocol provides a systematic approach to study the multifaceted processes of translating research findings into practice. Researchers from diverse backgrounds can follow this protocol to analyze how scientific knowledge is translated into effective health interventions, identifying critical factors that enhance or impede progress along the way. This framework can be used to assess the long-range impacts of successful translational science efforts as well as examples of incomplete implementation. The combined analysis of successes and failures should inform funding agencies regarding designing grant mechanisms and investing in future translational research.

With this protocol, we hope to generate excitement for the broad conduct of translational science case studies. Dissemination of this protocol is a first step toward enabling a novel, coordinated approach for this application of the science of translational science. While rigorous, well-researched case studies are individually valuable, the most exciting use of this protocol lies in cross-case analyses, to identify leverage points and exemplary practices, as well as theories of change developed for further empirical testing. To realize this potential, several complementary efforts should be pursued, including the establishment of dedicated publication outlets, coding schemas, and a case study archive. All of this will take time to realize fully, but the long-term payoffs will be worth the effort when we can demonstrate that insights from these case studies can be used to enhance translational research and speed the delivery of life-saving medical and health interventions.

## Supplementary material

For supplementary material accompanying this paper visit https://doi.org/10.1017/cts.2020.514. click here to view supplementary material
