# Supplementary material for: A protocol for retrospective translational science case studies of health interventions
Source: J Clin Transl Sci. 2020 Jul 22;5(1):e22. doi: 10.1017/cts.2020.514 (PMC8057422; doi:10.1017/cts.2020.514)
Supplement: Supplementary file 1 [file S2059866120005142sup.zip › S2059866120005142sup001.docx]

**Key Topics/Themes to address in an interview**

1. Original goal, problem, or hypothesis around which research is organized
2. Key developments in the research and development of intervention over time (including changes from original goal/hypothesis)
3. Critical support (funding, infrastructure, mentoring, resources, services, etc.)
4. Barriers and/or challenges that needed to be addressed to move the research forward
5. Facilitators/promoters that significantly helped with translation
6. Expected and unexpected outcomes/impacts
7. Involvement of non-academic groups (industry, community, advocacy groups, individuals)
8. Cross-/interdisciplinary collaboration/teams (role, nature of, extent of)
9. Critical technological/scientific tipping points that advanced the field
10. Major influences from/to other scientific fields that changed the trajectory
11. Future directions
12. Other key perspectives that should be considered

**Conducting interviews with key researchers**

Interviews will be semi-structured and tailored to the specific case being studied, with the goal of filling gaps in the information and expanding the story about the development of the intervention. Prior to the interviews, identify questions for the key researchers using the critical themes (above) and the broad interview questions (below) as *general interview guides*.

1. How did you get involved with this research? What problem were you addressing; or what was your hypothesis?
2. How did the research program evolve over time? What were the key translational markers? What specific research studies were seminal to the overall intervention?
3. What research supports/services were critical to the development of the research and how did those services advance the research?
4. What challenges/barriers did you have to address to complete this research successfully? At what points did the research stall, or show setbacks and why?
5. What worked well and why? Were there key facilitators/promotors to advancing the research?
6. Did you encounter unexpected events or findings (either positive or negative) that changed the direction of the research?
7. In the evolution of this research was there any important involvement of “outside groups” such as health advocacy groups, patient and family support groups, industry groups, etc. that played a role and how would characterize their role(s) and its effects?
8. What research teams were involved along the way? How did those research teams coalesce? What disciplinary backgrounds did team members have? How critical was it to the outcome to have a multidisciplinary team? To what extent did the research teams shift and change over time and why?
9. Were there technological or scientific breakthroughs that were critical to the progress of the research?
10. Were there major influences from/to other scientific fields that changed the trajectory of this research? To what extent have the key research findings led to research studies, applications, or interventions in other areas?
11. What are potential next steps in the research?
12. Are there other people we should we talk to in order to fill in missing parts of the story?
