# Supplementary material for: A protocol for retrospective translational science case studies of health interventions
Source: J Clin Transl Sci. 2020 Jul 22;5(1):e22. doi: 10.1017/cts.2020.514 (PMC8057422; doi:10.1017/cts.2020.514)
Supplement: Supplementary file 1 [file S2059866120005142sup.zip › S2059866120005142sup002.docx]

^[[1]](#endnote-1)^The table below provides an instructional framework for identifying information and underlying evidence to support translational research case studies as well as potential data sources. This information is intended to be illustrative, not comprehensive. In addition, certain categories of information and certain data sources will not be relevant to every case study.

| **Information Category** | **Information to Identify** | **Potential Data Sources** |
| --- | --- | --- |
| **Research** | **Research milestones spanning the translational research spectrum:**   1. **Basic/Fundamental** (e.g., studying the fundamental mechanisms of biology, health, disease, or behavior) 2. **Pre-clinical** (e.g., developing model systems, typically cellular, animal, or computational models, in which to study the etiology of disease as well as identifying & testing promising interventions) 3. **Clinical and health practice** (e.g., establishing the safety, efficacy, and effectiveness of interventional strategies) 4. **Clinical/health practice implementation & dissemination** (e.g., studying the level of adoption and/or factors which influence adoption of evidence-based interventions into routine clinical care or health practice for the general population) 5. **Public health** (e.g., assessing health outcomes at the population level to determine the effects of diseases and efforts to prevent, diagnose, and treat them) | *Publications (especially review articles), grant proposals, research funding databases, citations in documents (e.g., research articles, FDA approval packages, patent approvals, practice guidelines), & press releases/communications materials*  **Publications**  **PubMed:** <https://www.ncbi.nlm.nih.gov/pubmed/>  **Google Scholar:** <https://scholar.google.com/>  **AHRQ Systematic Review Data Repository:** <https://srdr.ahrq.gov/home/index>  **Cochrane Library:** <http://www.cochranelibrary.com/>  **Dimensions** (subscription required): <https://www.dimensions.ai/>  **Embase** (subscription required): <https://www.embase.com/#search>  **Web of Science** (subscription required): <https://webofknowledge.com/WOS>  **Scopus** (subscription required): <https://www.scopus.com/>  **Research funding and results databases:**  **NIH grants, publications, and patents (NIH RePORTer):** <https://projectreporter.nih.gov/reporter.cfm>  **Other U.S. Federal research funding (Federal RePORTer):** <https://federalreporter.nih.gov/>  **Dimensions** (subscription required): <https://www.dimensions.ai/>  **Approved drugs in U.S. (Drugs@FDA):** <https://www.accessdata.fda.gov/scripts/cder/daf/>  **US Patents:** <http://patft.uspto.gov/netahtml/PTO/search-adv.htm>  **Press releases/News**: <https://www.eurekalert.org/>  **Google:** [www.google.com](http://www.google.com)  **Stakeholder Interviews** |
|  | **Funding sources and other influential inputs** | *Funding acknowledgments in "milestone" publications, research funding databases, patent databases and related patent information*  **Research funding and results databases:**  **NIH grants, publications, and patents (NIH RePORTer):** <https://projectreporter.nih.gov/reporter.cfm>  **Other U.S. Federal research funding (Federal RePORTer):** <https://federalreporter.nih.gov/>  **Dimensions** (subscription required):  <https://www.dimensions.ai/>  **FDA Orange Book (Patents and exclusivity information of approved drugs):** <https://www.accessdata.fda.gov/scripts/cder/ob/index.cfm>  **US Patents:** <http://patft.uspto.gov/netahtml/PTO/search-adv.htm> (see especially the Assignee and Government Interest sections)  **PubMed:** <https://www.ncbi.nlm.nih.gov/pubmed/>  **Google:** [www.google.com](http://www.google.com)  **Stakeholder Interviews** |
| **Development (e.g., technology transfer, manufacturing, and scale-up)** | **Intellectual property (IP) & technology transfer/ licensing activities** | *Patents, industry reports, press releases*    **NIH RePORTER (Patents citing NIH funding):** <https://projectreporter.nih.gov/reporter.cfm>  **US Patents:** <http://patft.uspto.gov/netahtml/PTO/search-adv.htm>  **Press releases/News**: <https://www.eurekalert.org/>  **Derwent World Patent Index** (subscription required): <https://clarivate.com/products/derwent-world-patents-index/>  **Pharmaprojects** (subscription required): <https://pharmaintelligence.informa.com/products-and-services/data-and-analysis/pharmaprojects>  **Industry websites** & press releases  **Google:** [www.google.com](http://www.google.com)  **Stakeholder Interviews** |
|  | **Advances/innovations in manufacturing approaches and/or scale-up for commercial or public health use** | *Information to support overcoming issues or obstacles in making the intervention available at scale*  **Publications/PubMed:** <https://www.ncbi.nlm.nih.gov/pubmed/>  **Google:** [www.google.com](http://www.google.com)  **Industry websites** & press releases  **Stakeholder Interviews** |
| **Demonstrated utility to clinical practice** | **Established effectiveness of intervention in clinical/health practice** | *FDA approvals, publications (e.g., systematic reviews, clinical guidelines)*  **Drugs@FDA:** <https://www.accessdata.fda.gov/scripts/cder/daf/> [See especially the “Letters, Reviews, Labels, Patient Package Insert” information]  **FDA New Drug Approvals:** [[LINK](https://www.fda.gov/drugs/developmentapprovalprocess/druginnovation/default.htm)]  **AHRQ Systematic Review Data Repository:** <https://srdr.ahrq.gov/home/index>  **Cochrane Library:** <http://www.cochranelibrary.com/>  **UpToDate (Clinical Decision Tool – subscription required):** <https://www.uptodate.com/contents/search>  **Google:** [www.google.com](http://www.google.com)  **Stakeholder Interviews** |
| **Implementation & Dissemination** | **Evidence of *implementation*: successful integration of evidence-based interventions within a particular health care setting**  **Evidence of *dissemination*: widespread use of an evidence-based intervention by the target population** | *Inclusion in treatment guidelines, policies, clinical decision tools, insurer reimbursement decisions and claims data; peer-reviewed publications; industry reports; press releases*  **Pubmed:** <https://www.ncbi.nlm.nih.gov/pubmed/> [Use “Practice Guideline” as a MeSH term for Publication Type]  **AHRQ National Guideline Clearinghouse:** <https://www.ahrq.gov/gam/index.html>  **Other AHRQ databases:** <https://www.ahrq.gov/data/resources/index.html> [see especially Healthcare Cost and Utilization Project (HCUP) and Medical Expenditure Panel Survey (MEPS)]  **Press releases/News**: <https://www.eurekalert.org/>  **UpToDate (Clinical Decision Tool – subscription required):** <https://www.uptodate.com/contents/search>  **CMS Drug Spending Dashboards** (including Medicare and Medicaid): [[LINK](https://www.cms.gov/research-statistics-data-and-systems/statistics-trends-and-reports/information-on-prescription-drugs/index.html)]  **Other CMS databases:** <https://data.cms.gov/>  **Google:** [www.google.com](http://www.google.com)  **Stakeholder Interviews** |
| **Health impacts** | **Pre/post health outcomes of target population(s)** | *Population databases, patient registries, peer reviewed publications (including Phase IV studies), insurance claims data*  **Pubmed:** <https://www.ncbi.nlm.nih.gov/pubmed/>  **CDC’s Wide-ranging Online Data for Epidemiologic Research (WONDER):** <https://wonder.cdc.gov/>  **CDC’s FastStats:** <https://www.cdc.gov/nchs/fastats/default.htm>  **Other CDC Population health databases:** <https://www.cdc.gov/nchs/index.htm>  **AHRQ databases:** <https://www.ahrq.gov/data/resources/index.html> [see especially Healthcare Cost and Utilization Project (HCUP) and Medical Expenditure Panel Survey (MEPS)]  **CMS databases:** <https://data.cms.gov/>  **NCI Surveillance, Epidemiology, and End Results (SEER) database:** <https://seer.cancer.gov/>  **Stakeholder Interviews** |
|  | **Other: utility to follow-on innovations, health policy, health regulations, legal decisions, etc.** | *Publications, citations in policy, regulatory, and legal documents*  **Pubmed:** <https://www.ncbi.nlm.nih.gov/pubmed/>  **FDA New Drug Approvals:** [[LINK](https://www.fda.gov/drugs/developmentapprovalprocess/druginnovation/default.htm)]  **LexisNexis Academic legal literature database** (subscription required): <https://www.lexisnexis.com/hottopics/lnacademic/>  **Google:** [www.google.com](http://www.google.com)  **Stakeholder Interviews** |
| **Knowledge Impacts** | **Utility to follow-on research, methodological capabilities, and technology** | *Publications, awards (i.e., Nobel prize, Lasker award), other recognition (i.e., Science’s top discoveries)*  **Pubmed:** <https://www.ncbi.nlm.nih.gov/pubmed/>  **Press releases/News**: <https://www.eurekalert.org/>  **Google:** [www.google.com](http://www.google.com)  **Stakeholder Interviews** |
| **Other Societal impacts** | **Influence on industry/commercial activity** | *Publications, industry reports, press releases, FDA approvals*  **AHRQ databases:** <https://www.ahrq.gov/data/resources/index.html> [see especially Healthcare Cost and Utilization Project (HCUP) and Medical Expenditure Panel Survey (MEPS)]  **CMS Drug Spending Dashboards** (including Medicare and Medicaid): [[LINK](https://www.cms.gov/research-statistics-data-and-systems/statistics-trends-and-reports/information-on-prescription-drugs/index.html)]  **FDA New Drug Approvals:** [[LINK](https://www.fda.gov/drugs/developmentapprovalprocess/druginnovation/default.htm)]  **Market reports** (many examples; often need subscription)  **Industry websites**  **Pharmaprojects** (subscription required): <https://pharmaintelligence.informa.com/products-and-services/data-and-analysis/pharmaprojects>  **Google:** [www.google.com](http://www.google.com)  **Stakeholder Interviews** |
|  | **Cost-benefit of the intervention** | *Publications, insurance claims data, sales and other cost data*  **Pubmed:** <https://www.ncbi.nlm.nih.gov/pubmed/>  **AHRQ databases:** <https://www.ahrq.gov/data/resources/index.html> [see especially Healthcare Cost and Utilization Project (HCUP) and Medical Expenditure Panel Survey (MEPS)]  **CMS Drug Spending Dashboards** (including Medicare and Medicaid): [[LINK](https://www.cms.gov/research-statistics-data-and-systems/statistics-trends-and-reports/information-on-prescription-drugs/index.html)]  **Google:** [www.google.com](http://www.google.com) |

1. This framework has been adapted with input from MS Hamann, An Information Framework for Economic Analysis of Biomedical Research Outcomes. [unpublished, personal communication]. [↑](#endnote-ref-1)
