# Supplementary material for: A protocol for retrospective translational science case studies of health interventions
Source: J Clin Transl Sci. 2020 Jul 22;5(1):e22. doi: 10.1017/cts.2020.514 (PMC8057422; doi:10.1017/cts.2020.514)
Supplement: Supplementary file 1 [file S2059866120005142sup.zip › S2059866120005142sup003.docx]

# Expanded Protocol and Case Study Elements

### The Case Study Timeline

Complete case studies should include a timeline which serves as a key graphic for organizing, displaying, and communicating the case’s central information. The timeline progresses in phases along one or more pathways, punctuated by multiple markers/milestones that help to anchor the case study story. While timelines are typically linear, this is not meant to suggest that translation unfolds in a linear fashion; quite often translation moves backwards and forwards through different phases. However, the timeline is a universally understood device for visualizing temporal translational progression, e.g., “distance” between milestones.

Organizing elements of the timeline include:

1. **Start and End Points of Case Study:** Identifying the start and endpoints for any translational case will be subjective and may be challenging. Often, it is advantageous to first identify the endpoint, and then work backwards to converge on a logical start point.

Complete translational case studies link the chain of evidence from scientific observations to verifiable impacts of the specific intervention on health. The selected intervention should be applied currently in practice, e.g., in medical and public health settings, as opposed to research settings, and there should be evidence that the intervention improves health outcomes, increases life expectancy, and/or improves individuals’ quality of life. Ideally, the authors suggest the following guiding questions for selecting endpoints:

- Is there clear evidence that the intervention is in use, e.g., adoption of treatment guidelines?
- Is the intervention improving health outcomes?
- With the uptake of the intervention, are there concomitant changes in public health measures of disease burden?
- Are there indicators that the intervention has become a regular part of clinical practice (training curricula, medical board exams, certifications, or physician decision tools)?
- Has the intervention been incorporated into public health campaigns?
- Has the intervention informed changes in public policy regarding health-related issues?

The case study should address how far into practice the intervention has gone; this may include information about ways in which the intervention has been disseminated and/or implemented or otherwise “packaged” for scaling up. If the intervention is a drug, device, or biologic, evidence of translation into practice, such as post-clinical trials and post-FDA approval, is desirable and should be included, if available. The development and adoption of an intervention in clinical and community settings may continue to evolve far beyond the chosen endpoint of the case study.

Determining the most appropriate start point will take research and iteration. For complete translation, the start point is typically defined as the inception of the particular innovation being described in the case study and its association with a potential “target” such as a disease or diagnosis. The start point is likely to identify basic, foundational research knowledge that was essential for conceptualizing an effective intervention, perhaps going back decades or more in the research literature. Tracing backwards from the endpoint, the authors suggest utilizing several guiding questions to help converge on an appropriate start point:

- What/when was the first phenotypic understanding of the disease, disorder, or public health issue addressed by the intervention?
- What/when was the first appreciation of the underlying cause(s), disease mechanism(s), and/or the biological or behavioral “problem” to be addressed?
- What knowledge was essential to understand the scope of the disease/disorder/public health issue and who was impacted/vulnerable?
- What methodological and technological capabilities were required to test the intervention?

Depending on the focus of the case study, judgment must be used to determine how far back to trace the conceptual underpinnings of the intervention. It is often necessary to confine the scope of the case study to a manageable timeframe. Regardless of the chosen start/end points, they should be defined clearly so that readers may judge their suitability and validity.

1. **Progress Markers/Milestones:** Markers or milestones are integrally related to key events that occur during the translational process, including the start and endpoints of study. Markers are anchored on specific dates or represent a distinct time period and can be represented as points or intervals on a timeline. While not all markers discussed below will be applicable to all case studies, they should be chosen for their ability to help tell the story of the development and translation of the intervention. Different types of markers include: 1) major inputs, 2) key activities and events, and 3) major products or outputs. Inputs include resources (e.g., funding, key infrastructure used, transformational techniques applied, and community research resources), key individuals, and collaborations or partnerships. Key activities and events include major meetings, serendipitous events, policy changes, and clinical trial markers (e.g., IRB approvals, subject accrual, start and end of clinical trials). Examples of products and outputs are conference or meeting presentations, publications (one or more publications representing an emerging scientific consensus), investigational new drug approvals, clinical trials results (e.g., Phase 1, 2, 3, & 4), FDA new drug approvals, markers of commercialization (e.g., patenting, technology transfer, industry licensing), changes in clinical practice guidelines, behavioral change programs, changes in public health measures and any other evidence of hand-off to new entities along the translational timeline and the adoption of the intervention into practice.
2. **Translational Phases:** It is useful to group a complex translational timeline visually into general research phases. Numerous similar multi-phase schemes of translation have been proposed, but there is currently no universally accepted typology.[21] One model for these case studies is the NIH’s National Center for Advancing Translational Sciences’ translational phase model,[22] which includes the following research phases: Basic, Pre-clinical, Clinical, Clinical Implementation, and Public Health. Another useful translational research framework comes from the NIH’s National Institute of Environmental Health Sciences [23]; that framework was developed specifically to aid researchers in describing the evolution of their translational research in the area of environmental health. See Appendix B for suggested definitions and parameters for delineating research phases. Depending on the case, it may be necessary to apply a different translational model; however, whatever phase scheme is used should clearly identify the markers/milestones that demarcate each phase from the next.

### The Case Study Narrative

The second central element of the case study is the narrative, which provides a coherent summary that moves the reader through the translational science process, describing the major actors, themes, forces, pivotal events, and advances that had an influence on the translational process. The narrative should focus on describing *how and why* the intervention developed as it did, *how and why* the markers/milestones were achieved, as well as what challenges were encountered and how those challenges were addressed. These drivers of translation may arise directly from key documents and/or interviews with the central researchers and stakeholders (e.g., funders, community advocates, or practitioners). They may also arise indirectly through an analysis of the information gathered throughout the course of researching the case study. Given the interdisciplinary nature of translational research, the narrative should avoid discipline-specific jargon and instead should employ language that is easily understood by differing research and practice communities. In addition, consider the value of crafting the narrative for different target audiences, including a lay audience (see Section IV for a broader discussion of case study audiences and formats).

**The Case Study Narrative Key Elements**

1. **Health Problem & Relevance of the Intervention.** The case study narrative should begin with: a) background on the relevant disease(s), disorder(s), or public health challenge(s), including some measure(s) of burden to help communicate the scope of the problem; b) a description of the intervention; and c) a brief summary of the realized or potential impact or translational stage achieved.
2. **Key Events.** The key events are the scaffolding of the case study narrative and often correspond with the timeline progress markers/milestones, including the start and endpoints. The narrative is an ideal place to discuss considerations that affected the choice of start and endpoints, including potential alternatives. They describe the major activities that occurred over the course of the translational process. They can be singular events that occur at a single point or they can occur over a period of time. They constitute the heart of the chronological story, describing the sequence of integral events. Some of the candidates for key events in a case study include: major meetings and conferences; major discoveries and publications across the translational spectrum; key research and/or health practice interactions; clinical trials and trial milestones; research syntheses (meta-analyses, systematic reviews, guidelines); implementation and dissemination activities; and public health campaigns.
3. **Key People and Partnerships.** Over the course of translating an intervention from inception to impact, there are many individuals and groups who play important roles in either enhancing or inhibiting the translation. The case study should highlight the typically broad ecosystem of individuals and sectors involved in translating the research knowledge into health and societal applications. A description of the researcher(s) or research team(s) can include those who were central to the development, dissemination and implementation of the intervention, as well as other key individuals or groups, including industry, healthcare providers, healthcare organizations, policy makers, community stakeholders, professional societies, and patients/patient advocacy organizations. Determining who is discussed in a case study and why requires careful judgment and should be backed by objective evidence.

Additionally, there should be a description of how and why different individuals collaborated or interfaced with each other and what role such collaborations played in the development and implementation of the intervention. It is useful to understand the nature of key collaborations, such as how they were initiated, organized, resourced, and otherwise supported. For example, were the collaborations formal or informal? Were they initiated in a top-down or bottom-up manner? How did they integrate and benefit from multidisciplinary actors and approaches? What factors facilitated successful team dynamics and outcomes? To what extent did collaborators leverage previously existing partnerships, resources, or research relationships?

1. **Other Influencing Factors.** There are many other factors that can influence a translational research process, both in encouraging it and in hindering its progress. Influential factors operate at multiple levels of aggregation, from individual investigators, to research teams, to institutions, to communities, and beyond. Case studies should include descriptions of major facilitators, both expected and unexpected. Facilitators may include critical support (e.g., funding, infrastructure, mentoring, or other resources); federal or institutional policies; ground-breaking technologies, tools, and techniques; and knowledge or strategies borrowed from tangential lines of research that influenced the quality and speed of research outcomes. Case studies should also isolate major barriers or challenges, including failed or abandoned research directions, which presented substantial difficulties or problems that the researchers needed to address, as well as the ways in which those difficulties were overcome.

**Impacts.** The case study should provide evidence, and whenever possible, graphical displays that convey a current “snap-shot” of realized impacts. As noted in the introduction, there are several frameworks and metrics for assessing scientific research impact.[6, 8-14] One particularly extensive and useful community resource is the Becker Medical Library Model for Assessment of Research Impact.[10, 24]

Drawing from several frameworks, we describe three distinct categories of impacts: impacts on health, scientific knowledge, and society. Minimally, clear evidence of impacts on health are expected for any complete translational cases. (See Appendix B for a detailed description of diverse impacts that case study researchers could examine).

Case studies should describe health impacts, highlighting changes in health outcomes as well as the patient-level experience. Common health outcomes data include measures of disease incidence, prevalence, longevity, life expectancy, and Quality-of-Life measures. In the category of scientific knowledge, case studies may examine aspects such as knowledge spill-overs that have been instrumental in the emergence and growth of new fields, improved methodological and technological tools/applications, and award-winning contributions (e.g., Nobel Prizes and other forms of recognition for impactful research). Lastly, other societal benefits may include notable cost savings from improved intervention strategies, industry and commercial activity from research or medical products, new businesses created, economic growth (regional, national, or international), training of skilled health researchers and practitioners, improvements in science and health literacy, and international cooperation and capacity-building.

Impacts will rarely be fully attributable to the case of study; in most cases, they will be influenced by many additional causative and moderating factors not covered in the study. Therefore, case study researchers should take care to not “over-credit” their findings, while also providing compelling evidence that the central factors identified have played a critical, contributory role. In some cases, it may be valid and feasible to include analyses that project potential impacts that could accrue in the future (e.g., epidemiological, economic, or other modeling approaches to estimate what potential impacts could result).

1. **Further Developments:** Case study narratives may conclude with a description of how the research, dissemination, and/or implementation is currently progressing (or could progress); analysis of the remaining knowledge gaps and work that still needs to be done; and/or any important postscripts to the case study.
